# High-content high-throughput imaging reveals distinct connections between mitochondrial morphology and functionality for OXPHOS complex I, III, and V inhibitors

**DOI:** 10.1007/s10565-022-09712-6

**Published:** 2022-05-04

**Authors:** Wanda van der Stel, Huan Yang, Sylvia E. le Dévédec, Bob van de Water, Joost B. Beltman, Erik H. J. Danen

**Affiliations:** Division of Drug Discovery and Safety, Leiden Academic Centre of Drug Research, Leiden University, Einsteinweg, 55, 2333 CC Leiden, The Netherlands

**Keywords:** Mitochondria, Morphology, Machine learning, Membrane potential, ATP

## Abstract

Cells can adjust their mitochondrial morphology by altering the balance between mitochondrial fission and fusion to adapt to stressful conditions. The connection between a chemical perturbation, changes in mitochondrial function, and altered mitochondrial morphology is not well understood. Here, we made use of high-throughput high-content confocal microscopy to assess the effects of distinct classes of oxidative phosphorylation (OXPHOS) complex inhibitors on mitochondrial parameters in a concentration and time resolved manner. Mitochondrial morphology phenotypes were clustered based on machine learning algorithms and mitochondrial integrity patterns were mapped. In parallel, changes in mitochondrial membrane potential (MMP), mitochondrial and cellular ATP levels, and viability were microscopically assessed. We found that inhibition of MMP, mitochondrial ATP production, and oxygen consumption rate (OCR) using sublethal concentrations of complex I and III inhibitors did not trigger mitochondrial fragmentation. Instead, complex V inhibitors that suppressed ATP and OCR but increased MMP provoked a more fragmented mitochondrial morphology. In agreement, complex V but not complex I or III inhibitors triggered proteolytic cleavage of the mitochondrial fusion protein, OPA1. The relation between increased MMP and fragmentation did not extend beyond OXPHOS complex inhibitors: increasing MMP by blocking the mPTP pore did not lead to OPA1 cleavage or mitochondrial fragmentation and the OXPHOS uncoupler FCCP was associated with OPA1 cleavage and MMP reduction. Altogether, our findings connect vital mitochondrial functions and phenotypes in a high-throughput high-content confocal microscopy approach that help understanding of chemical-induced toxicity caused by OXPHOS complex perturbing chemicals.

**Supplementary Information:**

The online version contains supplementary material available at 10.1007/s10565-022-09712-6.

## Introduction

Mitochondrial malfunctioning plays a role in chemical induced toxicities and various diseases (Pacheu-Grau et al. 2018, Dykens and Will 2007). Disease-related mitochondrial defects, e.g., associated with Parkinson and Alzheimer disease, can be frequently traced back to mutations in genes involved in the major functions of the mitochondria (De Castro et al. 2010, Pacheu-Grau et al. 2018). Chemical-induced mitochondrial stress stimulates the cell to rearrange its mitochondrial pool and induces adaptive signaling responses in order to cope with reduced cellular functioning (Han et al. 2013). An inability to recover from the mitochondrial insult will induce apoptosis and/or necrosis to sacrifice the injured cell. Prolonged stress causing excessive cell death will result in organ failure (Bock and Tait 2020). Unraveling the concentration- and time-resolved relationships between mitochondrial perturbation and toxicity will provide useful information for chemical safety prediction and provide insight in adaptive and toxic responses to mitochondrial stress.

The type of chemical-insult dictates the type of cellular-mitochondrial responses. Chemicals may directly target key mitochondrial proteins such as oxidative phosphorylation (OXPHOS) complex components or cause cellular stress leading indirectly to damaged mitochondria. In response, mitochondria can trigger a mitochondrial unfolded protein response (UPRmt) to refold incorrectly assembled proteins (Münch 2018, Qureshi et al. 2017), activate mitochondrial biogenesis to increase size and numbers of mitochondria, adapt mitochondrial morphology and remodel OXPHOS complex components to increase OXPHOS capacity (Westermann 2010, Youle and Bliek 2012), and initiate mitophagy to dispose of malfunctioning mitochondria (Youle and Narendra 2011, Hamacher-Brady and Brady 2016). The specificity of these mitochondrial responses for certain types of stress could be used to understand and predict the type of chemical-induced perturbation.

Among the mitochondrial responses, morphological remodeling is one of the fastest and most flexible adaptation programs. Even under healthy conditions, mitochondria are highly dynamic organelles that constantly adapt their appearance based on the energy demand in various regions of the cells and facilitate mitophagy to get rid of undesired mitochondria (Westermann 2010, Giacomello et al. 2020). Different mitochondrial morphologies have been recorded in cell lines upon mitochondrial toxicant exposure, including antimycin, FCCP, oligomycin, and rotenone (Leonard et al. 2015, Koopman et al. 2005, 2006, Miyazono et al. 2018). These morphologies have been classified as puncta, rods, networks, small/large, etc. The presence of larger mitochondrial networks facilitates energy production, because of the increased interconnected membrane surfaces which can support OXPHOS. The formation of smaller mitochondria increases their mobility supporting energy demand in the outer areas of the cells and also simplifies degradation when desired (Westermann 2010).

Increasing and decreasing mitochondrial surface is achieved by changing the balance between fission and fusion (Seo et al. 2010). The machinery responsible for fission and fusion consists of a core group of GTPases: DRP1 is mostly responsible for fission and MFN1, MFN2, and OPA1 are responsible for fusion (Westermann 2010). Fission is achieved by dephosphorylation and polymerization of DRP1 at ER-mitochondrial sites. Fusion of mitochondria is initiated by the outer membrane protein MFN, which connects the outer membranes of two mitochondria and is subsequently followed by fusion of the inner membranes, which is facilitated by OPA1 (Giacomello et al. 2020). The exact trigger for the activation of the various fission and fusion related GTPases is not completely understood. Some of the GTPase members are activated via post-translational modification including cleavage and phosphorylation (Cribbs and Strack 2007, Jahani-Asl and Slack 2007, MacVicar and Langer 2016). Under healthy conditions, OPA1 is imported into the mitochondria and cleaved by the mitochondrial processing peptidase (MPP) into a long transmembrane protein (long OPA1/ L-OPA1) which supports the fusion process. Stress may activate proteases, like OMA1, that cleave OPA1 resulting in a shorter non-membrane bound form (short OPA1/ S-OPA1), which does not support fusion. Reported triggers that stimulate OPA1 cleavage and inhibit fusion include decreased ATP levels, a drop in mitochondrial membrane potential (MMP), heat shock, the loss of mitochondrial DNA, or mitochondrial swelling/opening of the mPTP pore (Anand et al. 2014, Baker et al. 2014, Ehses et al. 2009, Head et al. 2009, Consolato et al. 2018, Jang and Javadov 2020).

Here, we addressed whether changes in mitochondrial morphology can be coupled to key aspects of mitochondrial function in the context of chemical-induced toxicities. For this purpose, we systematically assessed the effect of distinct classes of OXPHOS complex perturbing agents in a time- and concentration-resolved manner. We established high throughput, high-content imaging methods and computational approaches to categorize mitochondrial morphology and monitor changes in ATP levels and MMP dynamics. This data was combined with an analysis of proteolytic cleavage of OPA1 as a key step in mitochondrial fusion. Lastly, we used gene silencing to address the impact of interfering with morphological adaptation on adaptation to mitochondrial stress. Our findings shed light on the dynamic relation between mitochondrial morphology and function in the context of chemical-induced stress derived from OXPHOS inhibition and create the opportunity to use mitochondrial morphology changes as a biomarker for the underlying mitochondrial perturbation.

## Materials and methods

### Chemicals

All tested chemicals were purchased via Sigma Aldrich (Germany): antimycin A (A8674), cyclosporin A (30024), FCCP (C2920), DCCD (D80002), diafenthiuron (31571), oligomycin (O4876), rotenone (R8875). The larger set of chemicals used to study difference between complex inhibitors was obtained via the JRC (Ispra Italy). Complex I inhibitors are capsaicin (Cat. No. M2028), deguelin (D0817), fenpyroximate (31684), pyrimidifen (35999), rotenone (R8875), and tebufenpyrad (46438). Complex II inhibitors are as follows: carboxin (45371), mepronil (33361), thifluzamide (49792. Complex III inhibitors are as follows: antimycin A (A8674), azoxystrobin (3167), cyazofamid (33874), picoxystrobin (33568), pyraclostrobin (33696). Stocks were created in dimethyl sulfoxide (DMSO) and stored at − 80 °C/ − 30 °C until further usage. Exposure medium was created at the day of usage and contained a max of 0.2% (v/v) DMSO. For all compounds, exposure times and concentration ranges used for analysis of mitochondrial morphology, MMP, ATP, and OPA1 cleavage were not associated with appearance of cell death as measured by PI staining (Suppl. Figure 1).

### Cell culture

All experiments were performed using HepG2 cells purchased from ATCC (American Type Culture Collection, Wesel, Germany). Cells were maintained in complete medium at 37 °C in a 5% CO2 humidified atmosphere. The complete medium consisted of Dulbecco’s modified Eagle’s medium (DMEM) (Fisher Scientific, 11504496), supplemented with 10% (v/v) FBS (fetal bovine serum), 25 U/ml penicillin and 25 μg/mL streptomycin (FBS; South American, Fisher Scientific, S181L-500 & PenStrep, Fisher Scientific, 15070–063). For experiments aiming to inhibit glycolysis, the medium was supplemented with 10 mM 2-deoxyglycose (2DG) (Sigma-Aldrich, D8375-5G) at the moment of exposure.

### Creation of HepG2 cell lines containing OPA-GFP or ATP-biosensors

OPA1 fused to GFP was introduced in HepG2 cells using the BAC technology previously optimized in our lab (Wink et al. 2017, Poser et al. 2008), i.e., a GFP-fusion spanning a large genomic fragment including the entire OPA1 gene (introns, exons, and its surrounding regulatory sequences) was integrated in the genome of the cells. HepG2 cells containing ATP-biosensors located in the mitochondria or cytoplasm were created using constructs kindly provided by Hiromi Imamura (Precursory Research for Embryonic Science, Japan Science and Technology Agency) (Imamura et al. 2009). Both for the BAC-GFP and the ATP biosensors constructs, 8 µg DNA was introduced using lipofectamine2000 (Fisher Scientific, 11668–027) into 2.000.000 HepG2 cells. Cells were kept on G418 (PAA/Brunschwig chemie, P31-011) selection starting with 0.25 mg/ml until confluency followed by 0.5 mg/ml until colony formation. Confocal imaging was used to select for GFP positive colonies (both for the OPA1-GFP and the ATP biosensor cell lines). The used OPA1-GFP clone was selected based on protein size in western blot. The used ATP biosensor clone was selected based on a homogenous expression.

### siRNA transfection

siRNAs targeting OPA1 (SMARTpool of 4 single siRNAs = MQ-005273–00-0002, siRNA1 = D-005273–01, siRNA2 = D-005273–02, siRNA3 = D-005273–03, siRNA4 = D-005273–04) were purchased from Dharmacon (US). Reverse transfections were performed in 24-well plates (Greiner, 662160) or 96-well screenstar black plate (Greiner Bio-One, 655892, 655866) using 40 or 50 nM siRNA and the transfection region INTERFERin (Polyplus, 409–50) in a dilution of 1:1000 or 1:1250. siRNA was diluted to 1 µM in 1 × siRNA buffer (Dharmacon, B-002000-UB-100). INTERFERin was diluted 50 × in SFM and antibiotic free medium. Upon 5-min incubation at RT, both solutions were mixed in a 1:3 (siRNA/INTERFERIN) ratio and incubated for 20–30 min at RT. Cell mixtures of 476,191 cells/well (24-well) or 23,000 cells/well (96-well) in medium containing FBS and antibiotics were mixed in a ratio of 6.25:1 or 5:1 to the siRNA-INTFERin mixture, respectively. Twenty four hours after transfection, the medium was refreshed and 72 h after transfection, chemical exposure followed by the desired readout was performed. Mock and kinase pool (KP) were used as controls. The KP is a mixture of 720 siRNA SMARTpools originating from the siGENOME human protein kinase library. It was used at the same total concentration as the other siRNA in the KP, resulting in extremely low concentrations of each individual siRNA in the KP, and therefore no knockdown.

### Confocal live cell imaging and analysis of MMP

Effects of chemical exposure on MMP was assessed using rhodamine123 (rho123) (sigma Aldrich, R8004) using the protocol described previously (van der Stel et al. 2020). Cells were seeded two days before exposure. At the day of exposure, cells were first co-stained with 200 ng/µl Hoechst (Life technologies, H1399) and 1 µM rho123 for 75 min, followed by a co-staining of 0.2 µM rho123 and 100 nM propidium iodide (PI) (Sigma-Aldrich, P4170) and exposure to the desired concentration of test chemical. Over a period of 24 h, the signal intensity of Hoechst (408 nm), rho123 (448 nm), and PI (561 nm) were monitored every hour. The nuclei were identified based on the Hoechst images and formed the basis for the assessment of the cytoplasm (defined as those pixels within a maximal distance of 10 pixels around the nucleus). Finally, the intensity of the rho123 was assessed in the cytoplasm, and cell death estimates were based on the fraction of nuclei that showed at least 10% of the pixels also positive for PI-staining. Part of the MMP data were used for development of a dynamic mathematical model and integration with RNA-seq data elsewhere (Yang et al. 2021, van der Stel et al. 2021).

### Confocal live cell imaging and analysis of ATP-biosensor

Effects of chemical exposure upon ATP fluctuations in the cytoplasm and mitochondria of HepG2 cells were assessed using the stably integrated ATP-biosensor constructs. The experiments were performed as previously described (van der Stel et al. 2020). Cells were seeded 2 days before chemical exposure. Imaging was performed every 5 min starting with two rounds of background measurement, followed by exposures and 2 h of imaging. The sensor was excited with the 408-nm laser and the FRET ratio was based on the emission at 408 and 488 nm. The obtained 408-nm images were used to determine the relevant cell area in which the 408 and 488 pixel intensities were determined. A more detailed description and illustrations are incorporated in previous work, and a part of the ATP data was also integrated in this prior work in combination with RNA-seq data (van der Stel et al. 2021).

### Confocal live cell imaging of mitochondrial morphology

Mitochondrial morphology was monitored using MitoTracker Red CMXRos (cell signaling, 9082) in a live confocal imaging setting. HepG2 cells were seeded with a density of 22,000 cells/well in a 96-well glass/screenstar black plate (Greiner Bio-One, 655892, 655866). Two days after seeding, cells were stained for 60 min at 37 °C with 200 ng/ml Hoechst 33342 (Life technologies, H1399) and 0.05 μM MitoTracker Red. After 60 min, the medium was replaced with complete DMEM containing 0.013 μM MitoTracker Red plus the desired concentration test chemical. The Hoechst and MitoTracker Red signal (respectively 408 and 561 nm) were monitored at 1 z-stack at the desired time points using in total 120 × zoom in a Nikon TiE2000 with perfect Focus System and xy-stage (Nikon, Amsterdam, The Netherlands). Zoom was created with (1) Plan Apo 60 × air objective (0.95 numerical aperture) plus 2 × digital zoom or (2) Apo LWD 40 × water objective (1.15 numerical aperture) plus 3 × zoom. The obtained image resolution was 1024 × 1024 pixels representing 0.1 µm/pixel and contained between 5 and 30 cells. Per assessed experimental conditions, with the exception of vehicle and the positive control oligomycin, there were 2 images included per biological replicate.

### Imaging data analysis for mitochondrial morphology data

#### Nuclei segmentation

CellProfiler (version 2.1.1 Broad Institute, Cambridge, USA) was used for the identification of nuclear objects. An in-house implemented segmentation module (Di et al. 2012) based on watershed algorithms designed for extra zoom pictures was used to segment nuclear objects from Hoechst intensity pictures, which were first equalized using a Gaussian filter.

#### Mitochondrial segmentation and feature quantification

Ilastik version 1.3.2post2 with a pixel classification workflow was used to identify MitoTracker Red positive pixels from background pixels (Berg et al. 2019, Sommer et al. 2011). The pixel classification workflow consists of: feature selection, training and classification. First, two filters were selected based on visual inspection: *Laplacian of Gaussian* (σ = 3.5) in 2D and *Difference of Gaussians* (σ = 3.5) in 2D. Secondly, fore- and background were annotated based on manual inspection of representative MitoTracker Red pictures. Next, a random forest classifier is used to classify all pixels into fore and background based on the selected features and annotations. Finally, the output from the pipeline is a “Simple Segmentation” picture, a binary picture with only fore- and background classification. Afterwards, custom python scripts were developed to utilize the Ilastik template in a headless mode, resulting in segmented mitochondrial objects in binary images. Subsequently, four features were used to describe object size and shape: area, perimeter, formfactor, and solidity. The area describes the size of an object in a number of pixels. The perimeter is the number of pixels at the boundary of an object. In the case of objects with a hole, the perimeter is the sum of both inner and outer boundary (i.e., the total length of the boundary between object and background). The formfactor is defined as $(4\times \pi \times \mathrm{area})/\mathrm{perimeter}$. The solidity is the quantification of the extent to which a shape is convex or concave. It is defined as $\mathrm{Area}/\mathrm{ConvexArea}$(ConvexArea = the area enclosed by a convex hull/minimal convex object which can enclose the object).

#### Fitting with a Gaussian mixture model (GMM)

A GMM was applied to classify the segmented mitochondria objects in an unsupervised manner based on the 4 object features. In this way, the objects were divided into two categories, referred to as “fragmented” or “fused.” The model was trained using all experimental images of cells exposed to various mitochondrial complex inhibitors and a vehicle control condition at time points ranging from 1 to 24 h. The GMM was implemented in python using 100 random starting values for the Gaussians. For each starting value, an expectation–maximization algorithm was utilized to minimize the negative log likelihood (− *log likelihood*) of the data. Finally, the model with the lowest –log likelihood was selected as the best fit. The class to which each mitochondrial object belonged was determined based on the probability for the two classes (i.e., the class having the highest probability was selected).

#### Statistical test

To compare control, compound exposure, and siRNA treatment, the ANOVA test combined with a Dunnett test was applied to analyze statistical significance.

#### Implementation

All data analyses were implemented in Linux with python 2.7 with packages scikit-learn for the GMM fitting and prediction, dash for interactive plotting and scipy.stats for statistical testing. Scripts available at: 10.5281/zenodo.6382996.

### ATPlite assay and analysis

ATP levels were assessed in cell lysate upon 2- and 24-h chemical exposure as previously described (van der Stel et al. 2020). Cells were seeded 2 days before exposure. At the third day, cells were stained with Hoechst for 2 h followed by chemical exposure. One hour before the end of the exposure period, the cells were imaged to monitor the Hoechst intensity. The Hoechst intensity images were used to segment the nuclear objects in every image and the total nuclear count per image was used to normalize further measurements. After the desired exposure period, the medium was replaced by Hanks’ buffer including 5 mM HEPES, 250 mM sucrose, 25 mM TRIS, 3 mM EGTA, 5 mM MgCl_2_, 5 mM succinate, and 5 mM glutamate (37 °C, pH 7.3) and for the mitochondrial fraction also supplemented for 30–45 s with 150 µM digitonin. ATP content was determined using the ATPlite 1 step Luminscence Assay reagent kit (PerkinElmer, 6016731). ATP data were integration with RNA-seq data elsewhere (van der Stel et al. 2021).

### Western blot

OPA1-GFP cleavage was monitored using western blot. Cells were plated at a density of 200,000 cells/well in 24 wells (Corning, 3524). Two days after seeding the cells were exposed to the test chemical for the desired timespan and lysed using direct lysis in 1 × times sample buffer (stock: 1% BromoPhenolBlue (Sigma, B0126-25G) in MQ plus few drops of NaOH to dissolve BPB) supplemented with 5% v/v β-mercaptoethanol (Fisher Scientific, 125472500). Samples were heat-denatured for 10 min at 95 °C before usage. Proteins were separated using SDS-PAGE on gels consisting of a running and stacking gel. The running contained per 10 ml: 5 ml MilliQ, 2.5 ml acrylamide (Bio-Rad, 1610158), 2.5 ml Tris (1.5 M Tris, with 10% SDS and *pH* = 8.8), 75 µl 10% APS (ammonium persulfate) (Sigma, A3678-25 g), and 15 µl TEMED (VWR, 17–1312-01). The stacking gel contained per 10 ml: 6.1 ml MilliQ, 1.3 ml acrylamide, 2.5 ml Tris (0.5 M Tris, with 10% SDS and *pH* = 6.8), 75 µl 10% APS and 15 µl TEMED. The proteins were blotted onto a PVDF membrane (Sigma, IPVH00010) using a wet transfer system. Membranes were incubated in 1% m/v milk in 1% TBS plus Tween20 (Boom, P2287-500 ml). Primary and secondary antibodies were diluted in TBS-Tween20, respectively 1:1000 and 1:2000. Primary antibodies included mouse-anti-GFP (1181460001, Roche, stock concentration = 0.4 mg/ml), mouse-anti-OPA1 (612606, BD Biosciences, stock concentration = 0.25 mg/ml), and mouse-anti-tubulin (T-9026, Sigma, stock concentration = 2.9 mg/ml). Secondary antibodies were goat-anti-mouse-HRP (115–035-003, Jackson, stock concentration = 0.4 m g/ml) and goat-anti-mouse-Cy5 (115–605-146, Jackson, stock concentration = 0.75 mg/ml). The horseradish peroxidase activity and Cy5 signal were detected using the Image Quant LAS4000 (GE HealthCare).

### Resazurin assay

The resazurin assay was conducted as previously described (van der Stel et al. 2020). Briefly, the supernatant was replaced, after the assessment of the mitochondrial membrane potential, with 44 µM resazurin dissolved in cell culture medium and incubated for 1.5 h in 5% CO_2_ at 37 °C. The colorimetric change upon resazurin reduction was assessed using 540 nm excitation and 590 nm emission.

## Results

### Analysis pipeline for quantitative categorization of mitochondrial morphology

**Fig. 1:**
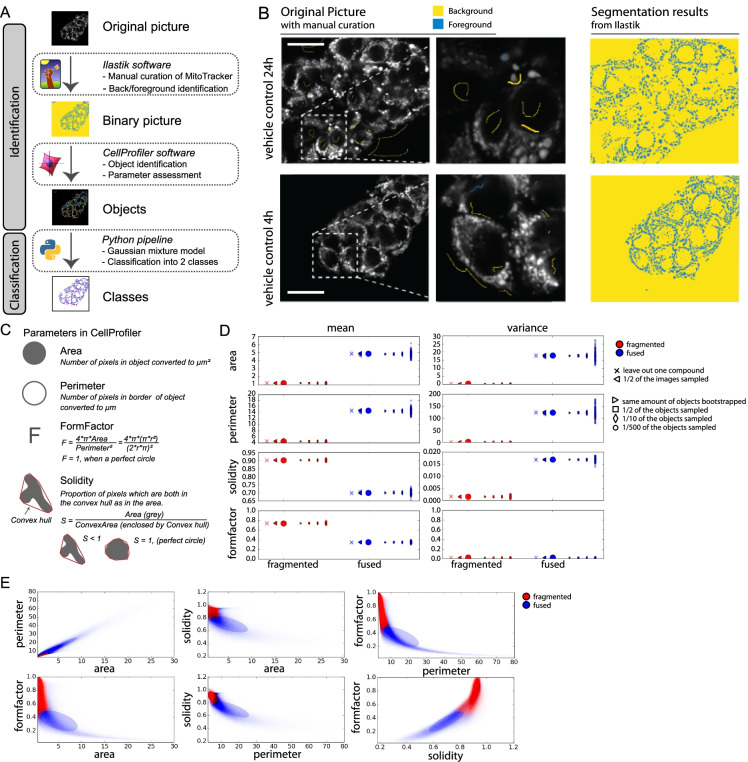
Quantification of mitochondrial morphology. **A** Schematic representation of the pipeline used to analyze mitochondrial morphology. The pipeline is designed to assess mitochondrial morphology in an unbiased approach using confocal images of mitochondria stained with MitoTracker Red in HepG2 cells. 1 The intensity images are converted to binary images using Ilastik software and based on manual curation, 2 various features are collected of the identified objects using CellProfiler, 3 these features are used to classify all mitochondria into 2 classes using a Gaussian mixture model (GMM) approach. **B** Manual curation of mitochondrial staining in the original MitoTracker Red images using Ilastik and the segmentation result from Ilastik based on the manual curation (yellow = background, blue = foreground). Scale bar represents 25.22 µm. **C** The selected parameters from CellProfiler software **D** Effect of the data size on the estimated mean and variance for the four morphological features in the GMM. Note that the fragmented mitochondria (red) have a much larger variance for area and perimeter than the fused mitochondria (blue), which is due to tha large variability of object size amogst fused mitochondria. **E** Scatter plot of two features (area, perimeter, solidity, formfactor) to describe mitochondrial morphology for mixture of two subpopulations. Red for fragmented mitochondria and blue for fused mitochondria. The ellipse kernels represent the Gaussian distribution for the two subpopulations (red for fragmented and blue for fused population. Mean and co-variance info are utilized to draw those ellipses. The contour lines indicating 95% confidence interval of the parameters

To assess the link between mitochondrial morphology and mitochondrial perturbation, we assembled an analysis pipeline to classify mitochondrial morphology into two morphology subclasses (“fragmented” and “fused”). To make this pipeline compatible with high-throughput data collection and include an unbiased morphology classification, we selected an unsupervised machine learning approach combined with open analysis software (Ilastik and CellProfiler) to segment and assess mitochondrial objects in living cells (HepG2) stained with MitoTracker Red and imaged using confocal microscopy (Fig. 1A).

Step one of the pipeline included identification of mitochondria from live confocal images in which mitochondria were stained with MitoTracker Red. Ilastik software was used to classify pixels of the MitoTracker Red images into fore- and background (Fig. 1B). In step two, CellProfiler was used to segment mitochondrial objects and quantify 4 features per object: area, perimeter, formfactor, and solidity (Fig. 1C). The third step consisted of an unsupervised learning approach using a Gaussian mixture model (GMM) to subdivide segmented mitochondrial objects based on the assessed features into two groups, i.e., fragmented and fused.

The parameter probability prediction from the GMM includes a mean and variance parameter per selected mitochondrial feature for both fragmented and fused mitochondria (Reynolds 2009) (Fig. 1D). To evaluate the effect of data size on GMM fitting, we monitored the effects on the mean and variance of the Gaussians when changing 3 aspects, i.e., the number of compounds, the number of images, and the number of objects. The effect of compound choice was studied using a leave-one-out strategy. The influence of the number of images was tested using 100 randomly selected subsets of the images (each time consisting of half of the images). Similarly, the effect of object number was estimated using only a fraction of all objects pooled from all conditions (using the fraction 1/2, 1/10, and 1/500 of all objects). This analysis showed that the data is sufficient to provide robust parameter estimates for the two mitochondrial classes (Fig. 1D). Moreover, visualization of the four considered features in two-dimensional scatter plots along with their estimated Gaussians demonstrated that the GMM clearly separates two classes within the feature space, which roughly distinguishes small, roundish mitochondria (referred to as fragmented), and large, irregularly shaped mitochondria (referred to as fused) (Fig. 1E).

### OXPHOS complex inhibitors disconnect mitochondrial morphology, membrane potential, and ATP production

**Fig. 2:**
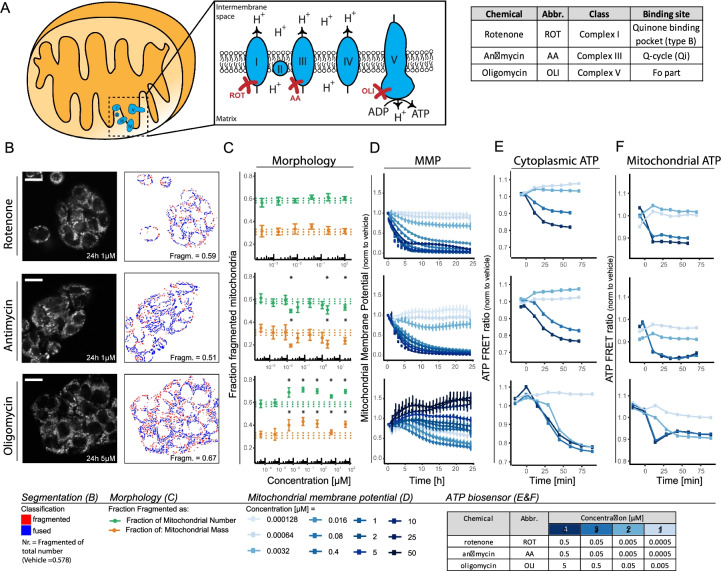
Quantitative analysis of ATP and MMP for 3 ETC complex inhibitors. **A** Schematic representation of the ETC in mitochondria showing the different complexes involved, the creation of a proton gradient, and the synthesis of ATP. Table includes information per compound concerning class and binding site. **B** Representative confocal imaging pictures of HepG2 cells stained with Hoechst (nuclei) and MitoTracker Red (mitochondria) and exposed for 24 h to 1 µM rotenone, 1 µM antimycin, or 5 µM oligomycin. The MitoTracker Red images are analyzed using the pipeline describe in Fig. 1, which resulted in a classification into two populations (red = fragmented, blue = fused). The quantified fraction of fragmented mitochondria as fraction of the total number of objects in the picture is depicted in the image. Scale bar represents 18.91 µm. **C** Fraction of mitochondria belonging to the class of fragmented mitochondria following 24-h exposure to vehicle control or a concentration range of oligomycin, rotenone, or antimycin. The two colors represent two methods: based on the number of identified objects (green) or incorporation of the identified object mass (orange). Data is represented as mean plus SE of three biological replicates. The dotted lines represent the average and SE of the vehicle treatment (0.2 v/v% DMSO). Asterisk represents a *p*-value < 0.05 for the comparison to the vehicle control. **D**, **E**, **F** Quantification of mitochondrial parameters upon exposure to a concentration range of rotenone, antimycin, or oligomycin. **D** MMP over time after exposure to 10 concentrations ranging from 0.000128 to 50 µM (data is normalized to DMSO and represented as a mean of 4 biological replicates ± SE); **E** cellular ATP levels over time upon exposure to 4 concentrations quantified using an ATP-biosensor (data is normalized to DMSO and from one replicate, additional biological replicates are shown in Suppl. Figure 2A); **F** mitochondrial ATP levels over time upon exposure to 4 concentrations quantified using an ATP-biosensor (data is normalized to DMSO and from one replicate, additional biological replicates are shown in Suppl. Figure 2A)

To be able to use mitochondrial morphology classification as a toxicity biomarker, it is important to understand the relationship between the readout and the biological effect. Alterations of mitochondrial morphology have previously been linked to distinct key functional properties including ATP levels and MMP (Anand et al. 2014, Baker et al. 2014, Ehses et al. 2009, Head et al. 2009, Consolato et al. 2018). We examined this relationship in the context of 3 known mitochondrial complex inhibitors: rotenone, antimycin, and oligomycin, targeting OXPHOS complexes I, III, and V, respectively (Fig. 2A).

Exposure to a concentration range of oligomycin induced an increase in fragmented mitochondria (Fig. 2B and C). Rotenone treatment did not result in a shift in the identified mitochondrial populations. Antimycin exposure induced a slight decrease in fragmented mitochondria (i.e., an increase in fused mitochondria) at high concentrations.

Next, we compared morphological responses to changes in MMP and ATP production. Rotenone and antimycin each triggered a concentration dependent decrease in MMP (Fig. 2D). By contrast, oligomycin stimulated a concentration-dependent increase of the MMP, which could be explained by blockage of ATP synthase, causing a proton gradient that is not used for ATP production (Symersky et al. 2012, Hong and Pedersen 2008, Yang et al. 2021).

With respect to changes in ATP production, rotenone, antimycin, and oligomycin each caused a concentration-dependent drop in ATP levels in the cytoplasm as measured by a cytoplasmic ATP sensor (Fig. 2D and E; Suppl. Figure 2A). Likewise, measurements using an ATP sensor localized to mitochondria showed that exposure to each of these chemicals resulted in a decrease in mitochondrial ATP and this drop was considerably faster than what was observed in the cytoplasmic compartment (Fig. 2D and F; Suppl. Figure 2A). As an alternative approach, ATP lite was used to measure overall whole cell ATP levels and ATP in the isolated mitochondrial fraction (Suppl. Figure 2B). Both demonstrated a similar concentration–response relationship for rotenone, antimycin, and oligomycin as observed with the ATP-biosensors, with the mitochondrial ATP fraction almost completely disappearing upon chemical exposure, when compared to the negative control.

In conclusion, while the three distinct OXPHOS complex inhibitors caused a similar decrease in mitochondrial ATP production, time and concentration-resolved analysis disconnected the responses at the level of mitochondrial morphology, MMP, and ATP production.

### OPA1 cleavage links OXPHOS complex inhibition to morphological adaptation

**Fig. 3:**
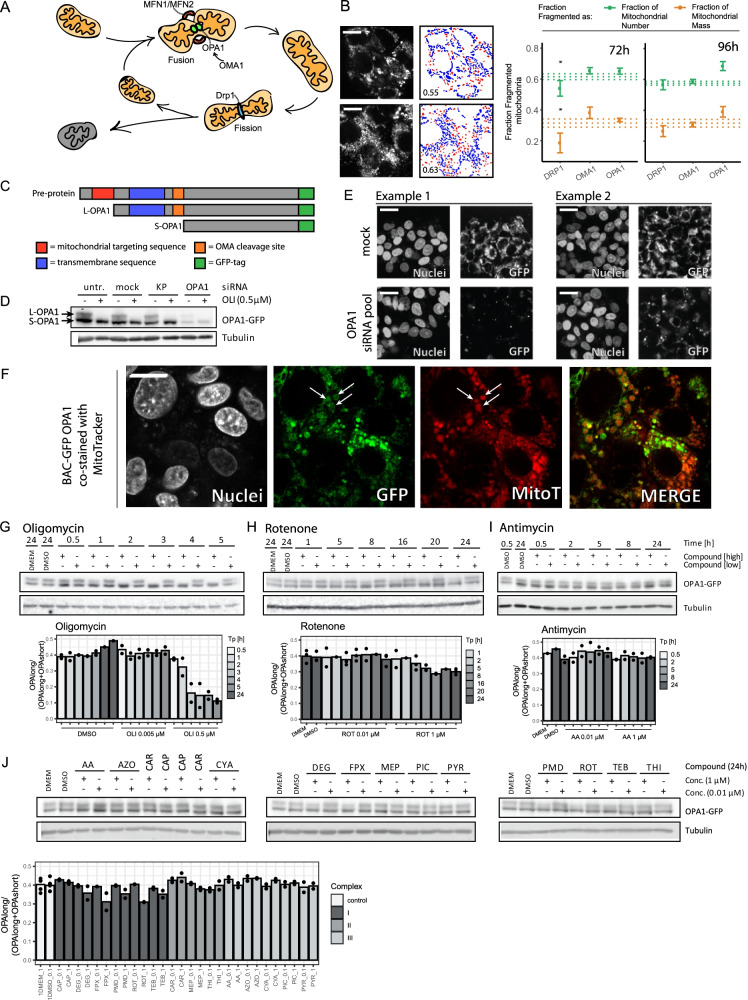
OPA1 cleavage correlates with mitochondrial fragmentation. **A** Scheme demonstrating the relationship between the process of fission and fusion and its major players. MFN1/MFN2 together with the membrane bound form of OPA1 are involved mitochondrial fusion. OMA1 de-activates OPA1 via cleavage. DRP1 together with the endoplasmic reticulum are responsible for mitochondrial fission. **B** Representative pictures of DMSO and OPA1 KD upon 96-h siRNA exposure. Scale bar represents 6.4 µm. In addition the visualization of segmentation plus fraction of fragmented mitochondria based on the total number of mitochondrial object (red = fragmented, blue = fused). The graphs depict the fraction of mitochondria belonging to the class of fragmented mitochondria 72 h or 96 h after silencing of *DRP1*, *OMA1*, and *OPA1*. The two colors represent two methods for determination of the fraction: based on the number of identified objects (green) or incorporation of the identified object mass (orange). Data is represented as mean plus SE of three or two biological replicates, respectively for 72 h and 96 h. The dotted lines represent the average and SE of the mock condition. Asterisks represent a *p*-value < 0.05 for the comparison to the vehicle control (no statistics were performed for the 96 h samples, because *N* = 2). **C** Scheme demonstrating the different forms of OPA1. 1 The pre-protein including mitochondrial targeting sequence and transmembrane sequence. 2 OPA1 is cleaved into L-OPA1 after entering the mitochondria. 3 L-OPA1 can be cleaved into S-OPA1 by OMA1 resulting in the loss of the transmembrane domain. The creation of a OPA1-GFP fusion protein using BAC technology resulted in a C-terminally tagged protein. **D** Western blot results for the BAC-GFP OPA1 cell line cultured in absence or presence of oligomycin (0.5 µM) in combination with the indicated siRNAs (untr, untreated; mock, transfection reagent only; KP, kinase pool; OPA1*,* siRNAs targeting *OPA1*). Samples were stained with anti-GFP and anti-tubulin (2nd and 3th replicate are included in Suppl. Figure 3A). **E** Representative confocal images of HepG2 BAC-GFP OPA1 cells treated with mock or siRNAs targeting *OPA1* and stained with Hoechst (nuclei). Scale bar represents 25.6 µm. **F** Representative confocal images of the HepG2 BAC-GFP OPA1 reporter cell line demonstrating the overlap of GFP signal and MitoTracker Red. Scale bar represents 7.23 µm. **G** Western blot results showing GFP and tubulin in HepG2 BAC-GFP OPA1 cells treated with 2 concentrations of oligomycin (0.005 or 0.5 µM) for 0.5, 1, 2, 3, 4, and 5 h. The bar graph depicts the mean of 2 biological replicates (replicates are individual dots). (Extra replicates are included in Suppl. Figure 3B and C). **H** Western blot results showing GFP and tubulin in HepG2 BAC-GFP OPA1 cells treated with 2 concentrations of rotenone (0.005 or 0.5 µM) for 1, 5, 8, 16, and 24 h. The bar graph depicts the level OPA1 long divided by total OPA1 and is the mean of 2 biological replicates (replicates are individual dots). (Extra replicates are included in Suppl. Figure 3D). **I** Western blot results showing GFP and tubulin in HepG2 BAC-GFP OPA1 cells treated with 2 concentrations of antimycin (0.005 or 0.5 µM) for 0.5, 2, 5, 8, and 24 h. The bar graph depicts the level OPA1 long divided by total OPA1 and is the mean of 2 biological replicates (replicates are individual dots). (Extra replicate is included in Suppl. Figure 3E). **J** Western blot results showing GFP and tubulin in HepG2 BAC-GFP OPA1 cells treated with 2 concentrations (0.1 or 1 µM) of 14 ETC complex inhibitors or controls for 24 h. The bar graph depicts the level OPA1 long divided by total OPA1 and is the mean of 2 biological replicates (replicates are individual dots) (Extra replicate is included in Suppl. Figure 3G)

To address the role of known mitochondrial morphology regulators in the occurrence of the fragmentation phenotype, we silenced the *DRP1*, *OMA1*, and *OPA1* genes and investigated the effect on mitochondrial morphology. Depletion of the fission protein DRP1 resulted in a decrease in the number of fragmented mitochondria at 72 h as compared to mock (Fig. 3B). OPA1 depletion caused an increase in mitochondrial fragmentation that was pronounced at 96 h after KD as was also observed for exposure to oligomycin (Figs. 2C and 3B). Depletion of the OPA1 regulator OMA1 did not result in a pronounced effect on mitochondria fragmentation. To further investigate the relationship between OPA1 and chemical-induced fragmentation, we developed an OPA1-GFP BAC fusion cell line (Fig. 3C, Suppl. Figure 3) and used OPA1 silencing to validate the specific detection of long and short OPA1 by GFP antibody in this cell line (Fig. 3D and E; Suppl. Figure 4A). The OPA1-GFP fusion protein localized in mitochondrial like structures in the cell and co-staining using a MitoTracker Red marker indeed demonstrated overlap (Fig. 3F).

To investigate how changes in the molecular machinery controlling fission/fusion related to the changes in morphology caused by OXPHOS complex inhibition, we first focused on oligomycin. The increased fragmentation induced by this compound (Fig. 2B and C) was indeed accompanied by a proteolytic cleavage leading to a loss of the L-OPA variant at 2 h (Fig. 3D, G; Suppl. Figure 4B and C). We next analyzed post-translational modification of OPA1 in response to rotenone and antimycin that each induced a similar decrease in ATP in the cytoplasm and mitochondria as observed with oligomycin (Fig. 2E and F; Suppl. Figure 2). Like oligomycin, rotenone exposure resulted in a reduction of L-OPA1, albeit less pronounced leaving a residual L-OPA1 expression perhaps explaining the lack of effect of rotenone on mitochondrial morphology (Figs. 2E and F and 3H; Suppl. Figure 4D). By contrast, but in agreement with its effect on mitochondrial morphology, antimycin exposure failed to induce L-OPA1 cleavage, despite the loss of ATP triggered by this compound (Figs. 2E and F and 3I; Suppl. Figure 4E). Together, these results indicated that mitochondrial fragmentation in response to OXPHOS complex inhibition correlated with L-OPA1 cleavage, but activation of this process cannot be explained by ATP depletion alone.

### Testing a larger panel of complex I and III inhibitors

We next compiled a larger set of chemicals all belonging to the classes of complex I and III inhibitors and supplemented this set with 3 complex II inhibitors. Our previous work demonstrated that all complex I and III inhibitors, except for capsaicin and cyazofamid, but not complex II inhibitors, attenuated basal oxygen consumption rate (OCR), and specificity for the assigned complexes was confirmed (van der Stel et al. 2020) (Suppl. Figure 4F). A 24-h exposure to concentrations above or around the OCR IC50 value for all active inhibitors did not result in a loss of L-OPA1. We only observed a minimal decrease of L-OPA1 upon exposure to complex I inhibitors deguelin, fenpyroximate, and rotenone (Fig. 3J; Suppl. Figure 4G). The absence of major reductions in L-OPA1 levels upon exposure to complex I and III inhibitors further indicated that a drop in ATP and OCR triggered by complex I and III inhibitors by itself cannot explain OPA1 cleavage. Rather, the complex V inhibitor oligomycin, in addition to ATP and OCR inhibition, must connect to OPA1 cleavage and mitochondrial fragmentation in another manner.

### Complex V inhibition also triggers OPA1 cleavage and mitochondrial fragmentation

**Fig. 4:**
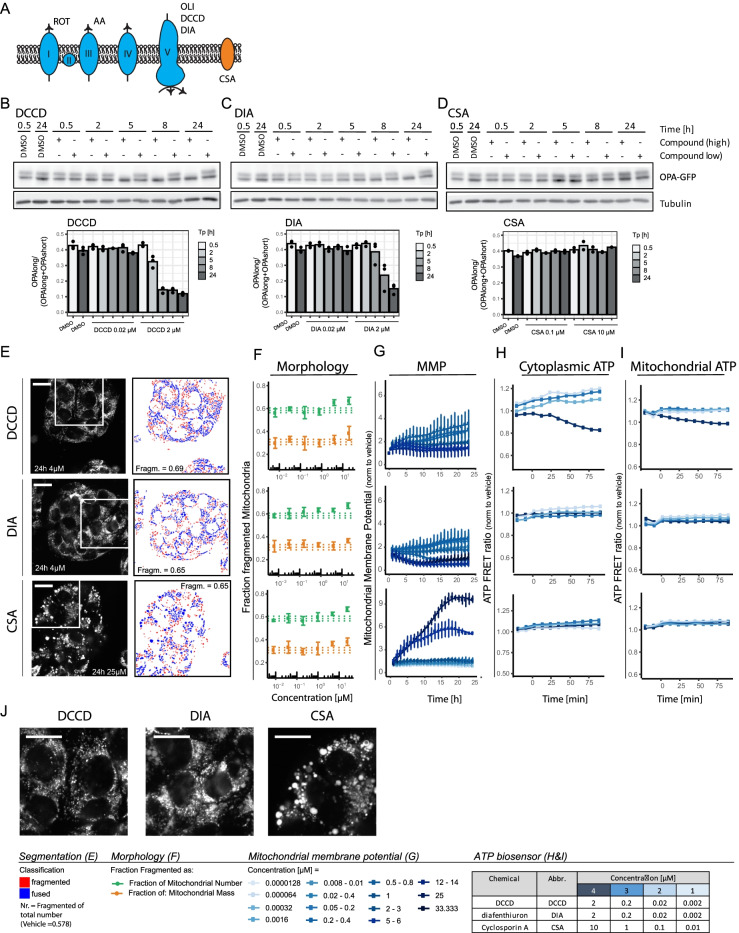
Impact of additional mitochondrial toxicants on mitochondrial function, morphology, and OPA1 cleavage. **A** Schematic representation of the molecular target of the different mitochondrial inhibitors used in this study. **B**, **C**, **D** Western blot results showing GFP and tubulin in HepG2 BAC-GFP OPA1 cells treated with 2 concentrations of **B** DCCD (0.02 or 2 µM), **C** DIA (0.02 or **D** 2 µM), or CSA (0.1 or 10 µM) for 0.5, 2, 8, and 24 h. The bar graph depicts the level OPA1 long divided by total OPA1 and is the mean of 3 (DCCD, DIA) or 2 9CSA biological replicates (replicates are individual dots). (Extra replicates are included in Suppl. Figure 4D). **E** Representative confocal images of HepG2 cells stained with Hoechst and MitoTracker Red (mitochondria) and exposed 24 h to vehicle control, 4 µM DCCD, 4 µM DIA, or 25 µM CSA. Segmentation pipeline demonstrating the distribution into two populations (red = fragmented, blue = fused). Bottom row demonstrates a zoom-in from top panel. The quantified fraction of fragmented mitochondria as fraction of the total number of objects in the picture is depicted in the image. Scale bar represents 19.33 µm. **F**, **G**, **H**, **I** Quantification of mitochondrial parameters after exposure to a concentration range of DCCD, DIA and CSA. **F** Fraction of mitochondria belonging to the class of fragmented mitochondria after chemical exposure. The two colors represent two methods: based on the number of identified objects (green) or incorporation of the identified object mass (orange). Data is represented as mean plus SE of three biological replicates. The dotted lines represent the average and SE of the DMSO condition. Asterisks represent a *p*-value < 0.05 for the comparison to the vehicle control; **G** mitochondrial membrane potential over time after exposure to 10 concentrations ranging from 0.000128 to 50 µM (data is normalized to DMSO and represented as a mean of 4 biological replicates ± SE); **H** cellular ATP levels over time after exposure to 4 concentrations quantified using cytoplasmic ATP-biosensor (data is normalized to DMSO and from one replicate, additional biological replicates are shown in Suppl. Figure 4A); **I** mitochondrial ATP levels over time after exposure to 4 concentrations quantified using mitochondrial-targeted ATP-biosensor (data is normalized to DMSO and from one replicate, additional biological replicates are shown in Suppl. Figure 4A). **J** Zoom in of Fig. 4E for DCCD, DIA, and CSA

While complex I, III, and V inhibition attenuated ATP levels and OCR, a distinguishing response to the complex V inhibitor oligomycin, as compared to the complex I and III inhibitors, was the increase in MMP in association with OPA1 cleavage and mitochondrial fragmentation. Two other chemicals known to inhibit complex V are DCCD and diafenthiuron (DIA) (Fig. 4A) (Hong and Pedersen 2008, Krieger 2001). As observed for active complex I and III inhibitors and the complex V inhibitor oligomycin, DCCD and DIA exposures resulted in an ATP decrease, although the response was delayed compared to other compounds (Fig. 4H and I and Suppl. Figure 5A and B; DIA = Suppl. Figure 5B). Like oligomycin, DCCD and DIA triggered an increase in MMP (Fig. 4G). Moreover, a 5-h exposure to 2 µM DCCD also resulted in a loss of L-OPA1 and exposure to 2 µM DIA resulted in a loss of L-OPA1 after 8 h (Fig. 4B and C; Suppl. Figure 5D). Finally, exposure to DCCD and DIA, similar to oligomycin, induced a concentration-dependent increase in the fraction of fragmented mitochondria (Fig. 4E and F). These findings showed that the reduction in ATP levels and OCR combined with an increase in MMP that specifically characterized complex V inhibition may trigger OPA1 cleavage and mitochondrial fragmentation.

### Increase in MMP by itself is insufficient to trigger mitochondrial fragmentation

To assess if the increase in MMP per se was sufficient to trigger mitochondrial fragmentation, we included cyclosporin A (CSA), which affects the MMP via a non-OXPHOS mechanism by blocking the mPTP pore resulting in a reduced exchange/leakage of protons between inner membrane space and mitochondrial matrix (Cassarino et al. 1998, Waldmeier et al. 2002, Mishra et al. 2019). Exposure to CSA resulted in an increased MMP without any effect upon ATP levels (Fig. 4F–H; Suppl. Figure 5A–C). CSA treatment did not affect OPA1 cleavage (Fig. 4C; Suppl. Figure 5D) but fragmentation appeared at high concentration (Fig. 4E). Notably, visual inspection of the mitochondrial morphology demonstrated that CSA also caused larger (perhaps swelled) objects rather that the smaller fragmented organelles observed with oligomycin, DCCD, or DIA exposure (Fig. 4J). These findings demonstrate that an increase in MMP, by itself, is not sufficient to trigger OPA1 cleavage. Note that although effects on mitochondrial morphology induced by CSA were classified as fragmented based on shape, this may require further study based on the larger size of the objects. We also tested FCCP, an uncoupler that directly disturbs the electron gradient causing loss of MMP and ATP levels (Suppl. Figure 6A–C), rather than inhibiting OXPHOS. This resulted in cleavage of OPA1 (Suppl. Figure 6D). However, MitoTracker staining became diffuse throughout the cell limiting the number of objects that could be selected for morphological analysis (Suppl. Figure 6E). Nevertheless, when these objects were analyzed, they were small and rounded, pointing to fragmentation (Suppl. Figure 6E). Altogether, this data indicates that interrelated morphological and functional mitochondrial responses observed for OXPHOS inhibitors cannot be translated to mitotoxicants acting through different mechanisms.

### OPA1 depletion similarly triggers mitochondrial fragmentation but does not enhance toxicity of OXPHOS complex inhibition

**Fig. 5:**
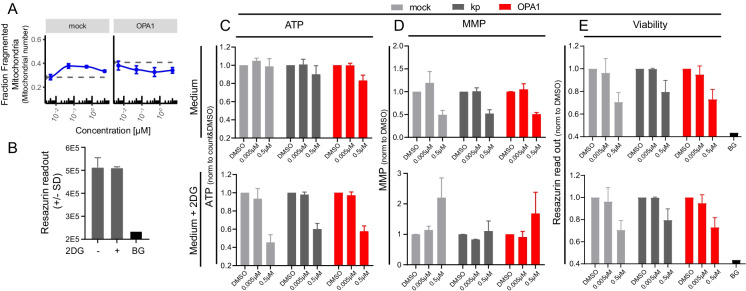
Effect of OPA1 silencing on mitochondrial morphology, viability, MMP, and ATP. **A** Fraction of mitochondria belonging to the class of fragmented mitochondria after 72 h mock or *OPA1* siRNAs followed by 24 h oligomycin (0.005, 0.05, 0.5, and 5 µM) (blue line) or vehicle (0.2 v/v% DMSO) exposure (dotted line). Data is represented as mean fragmented fraction plus SE (based on number of objects identified) of three biological replicates. **B** Quantification of viability based on resazurin conversion for medium with/without 2DG 96 h after KD. Data is a representation of mean plus SD of 3 replicates (BG, background signal). **C–E** Quantification of mitochondrial parameters in cells treated 72 h with indicated siRNAs (see legend Fig. 5B) followed by exposure to oligomycin (0.005, 0.5 µM) in medium with/without 2DG. Data is represented as a mean of 3 biological replicates plus SD). **C** Total cellular ATP levels quantified using ATPlite assay after 5-h oligomycin exposure. **D** Mitochondrial membrane potential at 24-h oligomycin exposure. **E** Quantification of viability based on resazurin conversion after 24-h oligomycin exposure

As our findings indicated selective OPA1 cleavage (causing a loss of OPA1 function) in the subset of OXPHOS complex inhibitors triggering mitochondrial fragmentation, we tested the effect of OPA1 depletion by itself. Silencing *OPA1* resulted in an increase in fragmented mitochondrial objects that was similar to the level induced by oligomycin (Fig. 5A; compare dashed line in right panel to blue curve in left panel). Combining OPA1 depletion with oligomycin treatment did not further increase mitochondrial fragmentation.

We assessed whether OPA1 depletion by itself led to toxicity under regular cell culture conditions and in presence of 2-deoxyglucose (2DG) to enforce a strict dependence on mitochondrial respiration. 2DG was added to the medium at a level that did not affect cellular viability (Fig. 5B). OPA1 depletion did not affect ATP levels, MMP, or viability by itself under control or 2DG conditions (Fig. 5C–E). Moreover, the reduction in ATP, the increase in MMP, and the reduced viability observed with 0.5 µM oligomycin were not amplified by OPA1 depletion.

Taken together, our findings point to a combination of reduced ATP levels and OCR with increased MMP as the trigger for OPA1 cleavage and mitochondrial fragmentation that is observed with exposure to OXPHOS complex V inhibitors. Instead, complex I and III inhibitors showing reduced ATP, OCR, and MMP do not trigger mitochondrial fragmentation or OPA1 cleavage. OPA1 silencing is sufficient to trigger a similar mitochondrial fragmentation but this level of fragmentation cannot be assigned to a (mal)adaptive response as OPA1 silencing by itself does not affect viability and fails to (de)sensitize cells to OXPHOS complex inhibition. Lastly, the interrelated morphological and functional mitochondrial responses observed for OXPHOS inhibitors, cannot be translated to mitotoxicants acting through different mechanisms.

## Discussion

In this work, we assessed the interconnectivity of mitochondrial morphology, MMP, and ATP production in the context of chemical exposure to distinct OXPHOS complex inhibitors (see Suppl. Figure 7 for an overview). Our results demonstrate that the different mitochondrial morphologies, fused or fragmented organelles as induced by treatment with different OXPHOS inhibitors, can be reproducibly quantified in a high-content, high-throughput, confocal imaging setup. We identified that changes in ATP or MMP alone could not explain the observed chemical-induced changes in mitochondrial morphology. We show how different OXPHOS inhibitors can be distinguished based on distinct, interrelated morphological, and functional morphological responses. Our data indicates that the integration of multiple mitochondrial features in a time- and concentration-dependent approach can improve mechanism-based characterization of mitochondrial toxicants.

Changes in mitochondrial morphology have been linked to alterations in MMP, production of ATP, and other mitochondrial processes (Anand et al. 2014, Baker et al. 2014, Ehses et al. 2009, Head et al. 2009, Consolato et al. 2018). We combined real-time confocal microscopy analysis of mitochondrial morphology, MMP, and ATP levels to assess their temporal relationships in the context of exposure to concentration ranges of OXPHOS complex inhibitors. We used a set of mitochondrial toxicants with a well-defined mode-of-action. The observed mitochondrial objects were quantified based on size and shape descriptors used in earlier studies: area, perimeter, formfactor, and solidity (Bondi et al. 2016, Irobi et al. 2012, Rodríguez-Martín et al. 2016). The use of an unsupervised machine learning pipeline enabled us to separate fragmented and fused objects based on the measured object features. We specifically aimed to develop methodology that was compatible with higher throughput analysis of multiple compounds tested at multiple concentrations and a range of timepoints. Inevitably, this comes at a cost: image resolution is lower. Nevertheless, we can reproducibly distinguish OXPHOS inhibitors that trigger enhanced mitochondrial fragmentation from OXPHOS inhibitors that do not. This is corroborated by the fact that OXPHOS inhibitors triggering fragmentation also trigger OPA1 cleavage whereas OXPHOS inhibitors that do not trigger fragmentation do not trigger OPA1 cleavage. On the other hand, we observe that interpretation is not straightforward for other classes of mitotoxicants that do not act as OXPHOS inhibitors.

Others have reported a correlation of mitochondrial fission with the perimeter and solidity of the detected objects (Westrate et al. 2014). The larger variance observed in our data for the size descriptors (area and perimeter) compared to the shape features (solidity and formfactor) indicates that fragmented and fused mitochondria are more easily distinguished by their shape than by their size. The fact that CSA is classified as “fragmented” while also rounded objects of larger size are observed indicates that swelling, which may be caused by mPTP opening triggered by this compound, may not be properly discriminated from fragmentation by our methodology. Because of the used set of mitochondrial toxicants, the established model is biased towards identification of smaller objects and would benefit from a more diverse set of treatments including those enhancing fusion or inhibiting fission. As an alternative, supervised learning can be employed for mitochondrial classification purposes (Leonard et al. 2015, Peng et al. 2011). However, supervised learning is based on example mitochondrial objects and requires high-resolution images for identification of detailed object features. Our approach is applicable to lower resolution images and therefore fits more readily to high-throughput approaches. This creates the opportunity to systematically screen large sets of chemicals for their effects upon mitochondrial morphology.

This setup also allowed us to combine high-throughput analysis of mitochondrial morphology with other, functional mitochondrial readouts developed for the same imaging format. For this purpose, we focused on MMP using dyes depending on MMP to accumulate in mitochondria and on the production of ATP using ATP biosensors localized in mitochondria or in the cytoplasm. A decrease in MMP (Jones et al. 2017) or a decrease in ATP (Jones et al. 2017) has been reported to drive OPA-mediated changes in morphology. Our findings show that the relationship between MMP, ATP production, and mitochondrial morphology is more complex; complex V inhibitors causing an increase in MMP trigger OPA1 cleavage and mitochondrial fragmentation. Importantly, increasing MMP alone by blocking the mPTP pore does not lead to OPA1 cleavage and mitochondrial fragmentation. Instead, a combination of MMP increase with a reduction in ATP production and OCR is what characterizes the OXPHOS complex V inhibitors that trigger mitochondrial fragmentation. Indeed, this subset of OXPHOS complex inhibitors also triggers OPA1 cleavage. Moreover, OPA1 silencing is sufficient to trigger a similar mitochondrial fragmentation. These findings suggest a delicate control of the upstream regulators of OPA1 cleavage such as the protease OMA1, which acts as a sensor for mitochondrial stress (Baker et al. 2014). Notably, our experiments using CSA, which blocks the mPTP pore, or those using FCCP, which triggers transport of protons across the mitochondrial inner membrane, thereby interfering with the proton gradient, indicate that our findings showing that different OXPHOS inhibitors can be distinguished based on distinct, interrelated morphological, and functional responses cannot be translated to compounds affecting mitochondria through other mechanisms.

We find no evidence that mitochondrial fragmentation associated with OPA1 cleavage represents a (mal)adaptive response. First, OPA1 silencing in our model system, which causes a similar level of mitochondrial fragmentation as observed with the subset of OXPHOS complex inhibitors, by itself does not affect ATP production or cell viability. Secondly, OPA1 silencing does not (de)sensitize cells to OXPHOS inhibition. In other in vitro and in vivo systems, enhanced mitochondrial fragmentation has been associated with pathological phenotypes and cell death (Zorov et al. 2019) and overexpression of OPA1 or another fusion related protein, MFN resulted in protection against mitochondria-related toxicities and disease (Hwang et al. 2014, Alaimo et al. 2014, Patten et al. 2014, Civiletto et al. 2015). Vice versa, overexpression of the fission protein DRP1 worsened rotenone toxicity in drosophila (Hwang et al. 2014) while pharmacological inhibition of Drp1 with mdivi protects cells against oxygen–glucose deprivation (Grohm et al. 2012, Tian et al. 2014). The reason for the apparent discrepancy between these studies and our own finding is not clear but the modest shift from fusion to fission achieved by OPA1 silencing or by OXPHOS complex inhibition in our study may be tolerated while strong overexpression, a full KO, or pharmacological inhibition of fusion/fission proteins may impact on viability.

In addition to providing new insight in the relationship between different responses to OXPHOS complex inhibition (mitochondrial morphology, MMP, cellular and mitochondrial ATP, cell viability), this study sets the stage for chemical screening using multi-parameter microscopic readouts. The next-generation chemical risk assessment paradigm stimulates the use of mechanism-driven studies in which biology-relevant parameters are quantified using sophisticated unbiased assessment methods and integrated into networks of prediction models (Krewski et al. 2014). The data obtained in this study fits this type of risk assessment, when combined with for instance ordinary differential equations (ODEs) to describe the time and concentration dependency of the ATP and MMP alterations (Yang et al. 2021, Daun et al. 2008). Such modeling efforts will result in threshold values describing the concentration, moment in time, or the effect size at which one event will trigger the next. This process will benefit from the incorporation of single cell measurements describing multiple events in one cell, which will enable establishment of relationships between ATP, MMP, and distribution of mitochondrial objects. The time- and concentration-resolved multi-parameter data such as generated in this study can be applied in screening of a wider range of chemicals and provide quantitative data for adverse outcome pathway (AOPs) (Leist et al. 2017).

## Supplementary Information

Below is the link to the electronic supplementary material.

Supplementary file1 (PDF 6958 KB)

## Data Availability

The data generated during the current study are available from the corresponding author on reasonable request.

## Code availability

Scripts used for the assessment of mitochondrial morphology classes are available at https://doi.org/10.5281/zenodo.6382996.
