# Supplementary material for: High-content high-throughput imaging reveals distinct connections between mitochondrial morphology and functionality for OXPHOS complex I, III, and V inhibitors
Source: Cell Biol Toxicol. 2022 May 4;39(2):415–33. doi: 10.1007/s10565-022-09712-6 (PMC10247858; doi:10.1007/s10565-022-09712-6)
Supplement: Supplementary file 1 — Supplementary file1 (PDF 6958 KB) [file 10565_2022_9712_MOESM1_ESM.pdf]

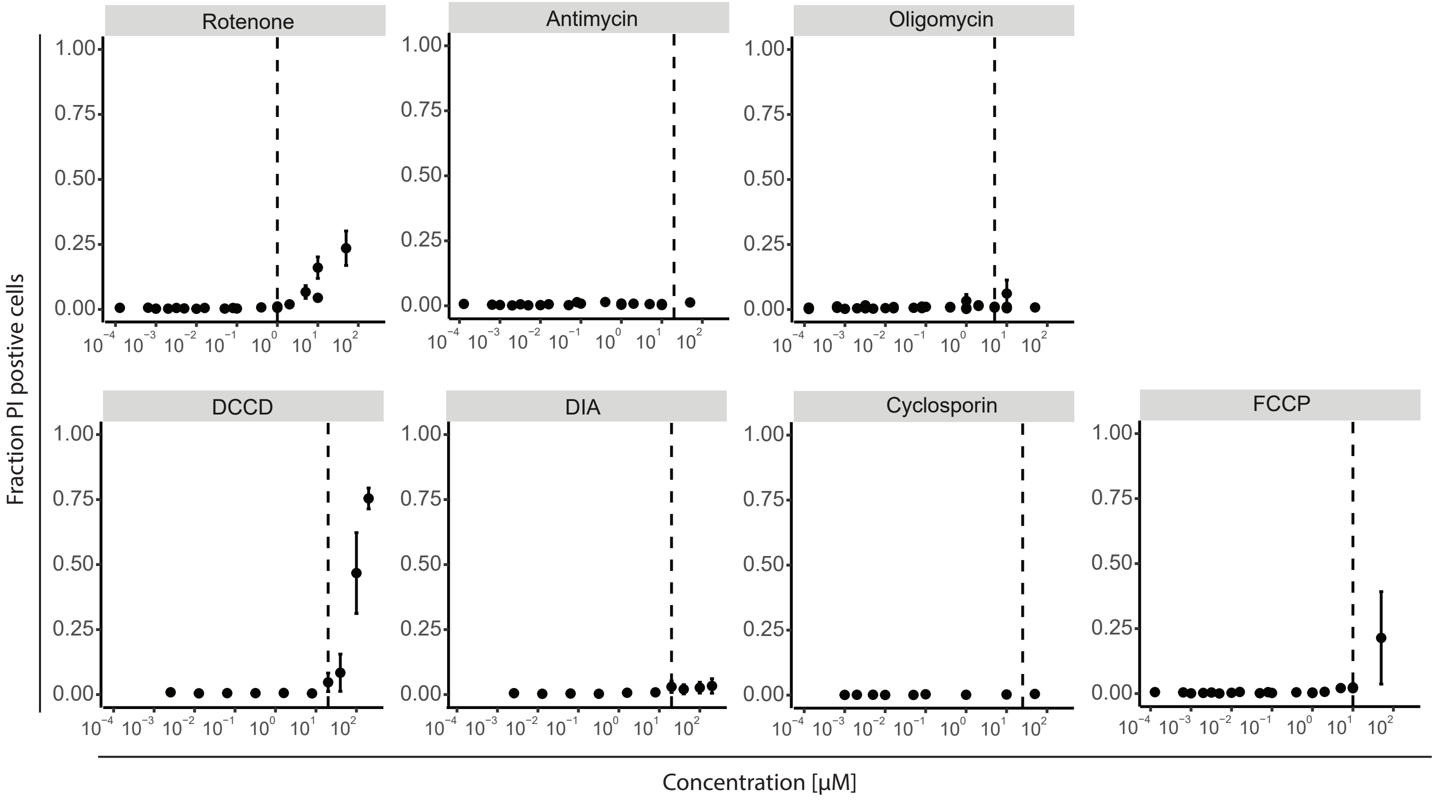

Supplementary figure 1

A

Chemical	Abbr.	Concentration [µM]			
		4	3	2	1
rotenone	ROT	0.5	0.05	0.005	0.0005
antimycin	AA	0.5	0.05	0.005	0.0005
oligomycin	OLI	5	0.5	0.05	0.005

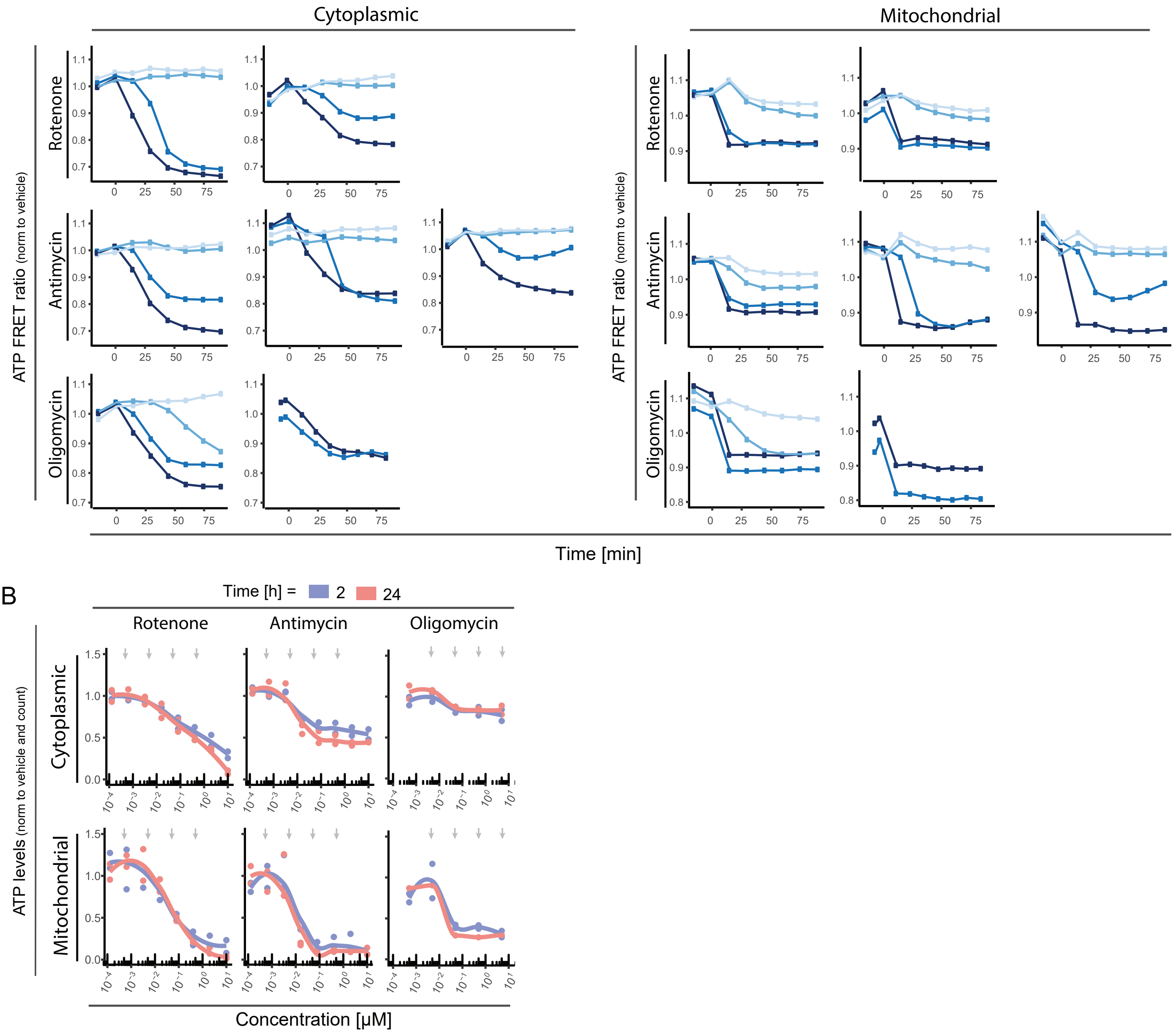

Supplementary Figure 2

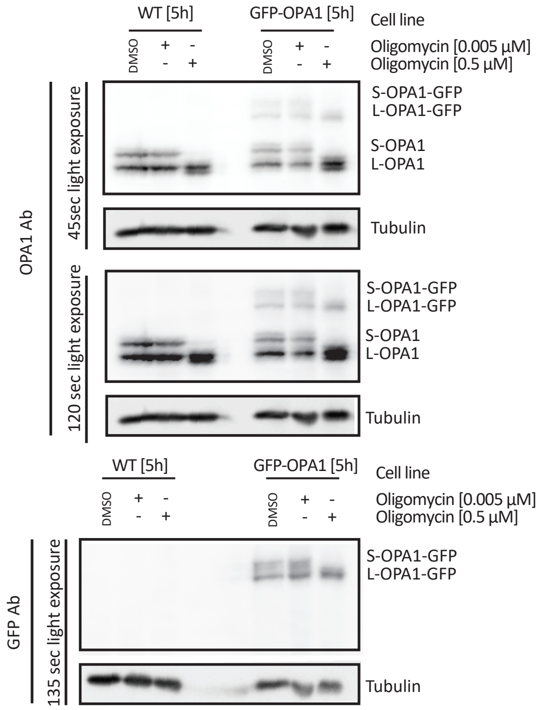

Supplementary Figure 3

A

B

C

D

E

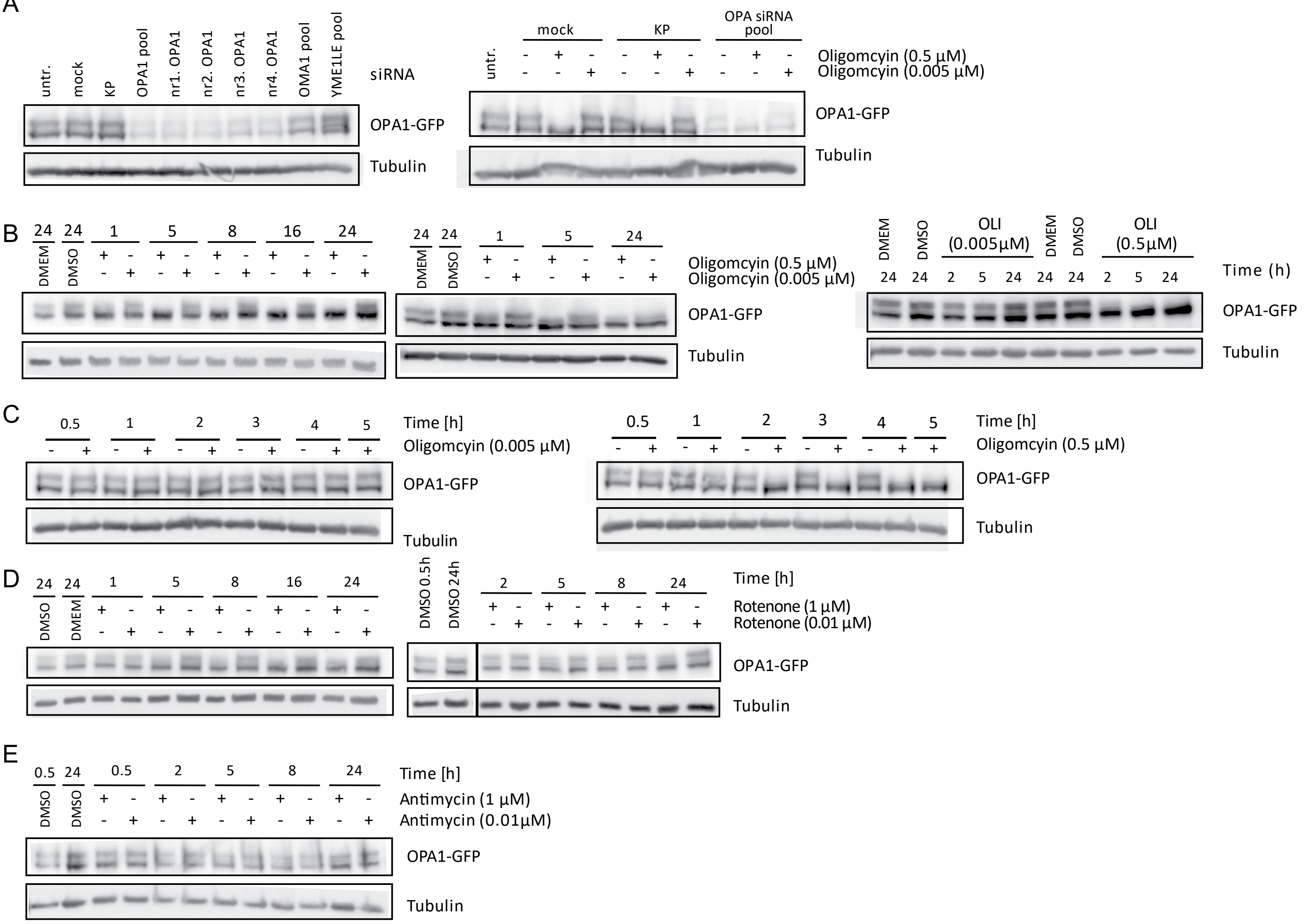

F

Compound	Abbr.	Complex	OCR Basal Intact cells IC50 [μM]	OCR Basal Permeabilised cells
Capsaicin	CAP	CI	> 10	CI
Deguelin	DEG	CI	0.676	CI
Fenpyroximate	FPX	CI	0.676	CI
Pyrimidifen	PMD	CI	0.079	CI
Rotenone	ROT	CI	0.240	CI
Tebufenpyrad	TEB	CI	0.851	CI
Carboxine	CAR	CII	> 10	CI > CI/CIII
Mepronil	MEP	CII	> 10	CI > CI/CIII
Thifluzamide	THI	CII	> 10	CI/CIII
Antimycin A	AA	CIII	0.028	CI = CIICIII
Azoxystrobin	AZO	CIII	> 10	CI = CIICIII
Cyazofamid	CYA	CIII	> 10	CI = CIICIII
Picoxystrobin	PIC	CIII	2.344	CI = CIICIII
Pyraclostrobin	PYR	CIII	2.089	CI = CIICIII

G

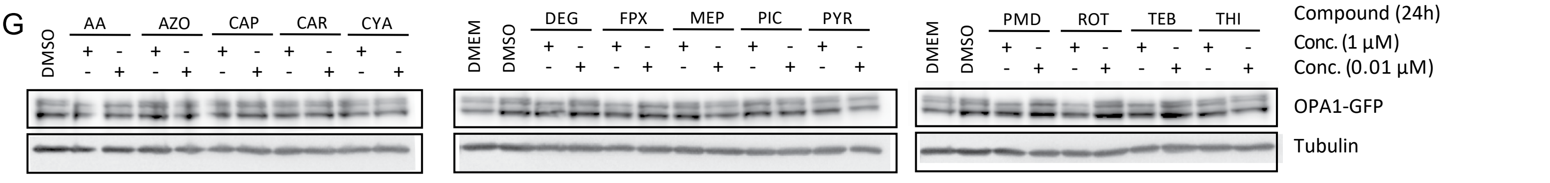

Supplementary Figure 4

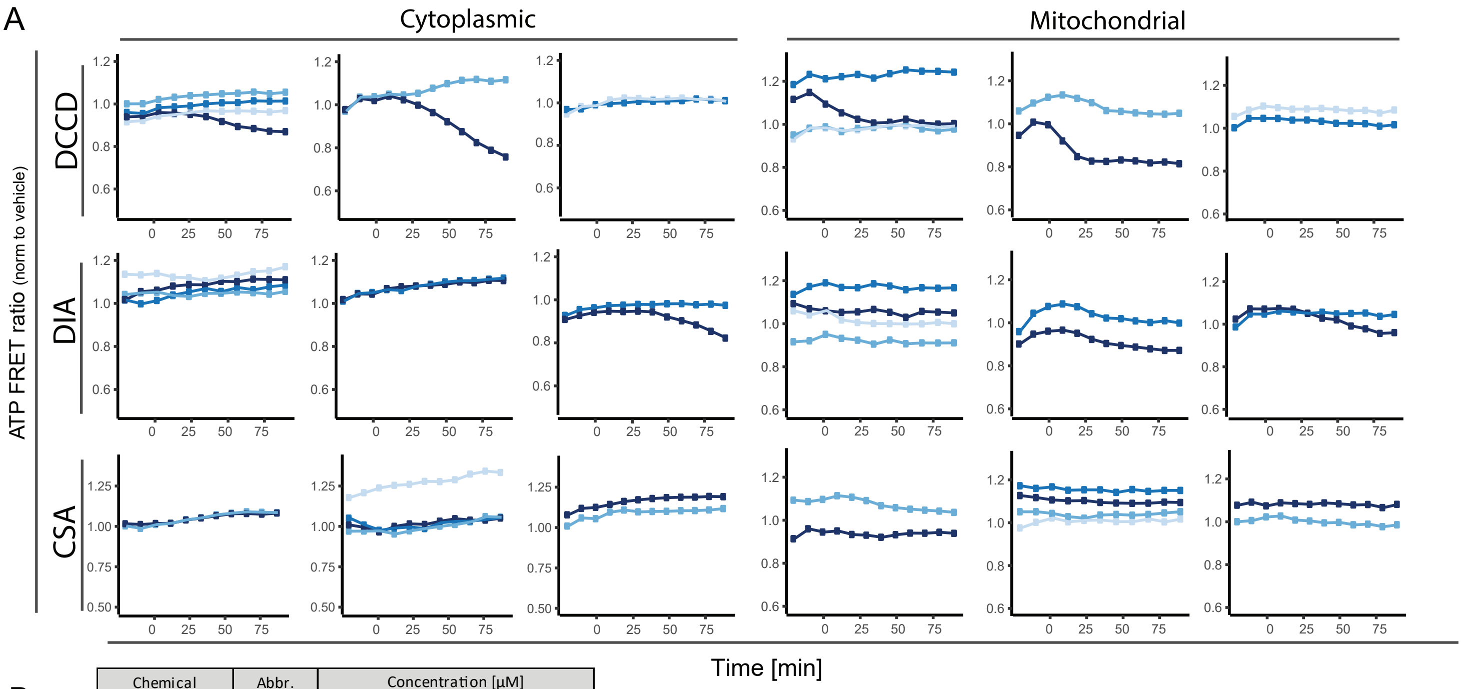

B

Chemical	Abbr.	Concentration [µM]			
		4	3	2	1
DCCD	DCCD	2	0.2	0.02	0.002
diafenthiuron	DIA	2	0.2	0.02	0.002
Cyclosporin A	CSA	10	1	0.1	0.01

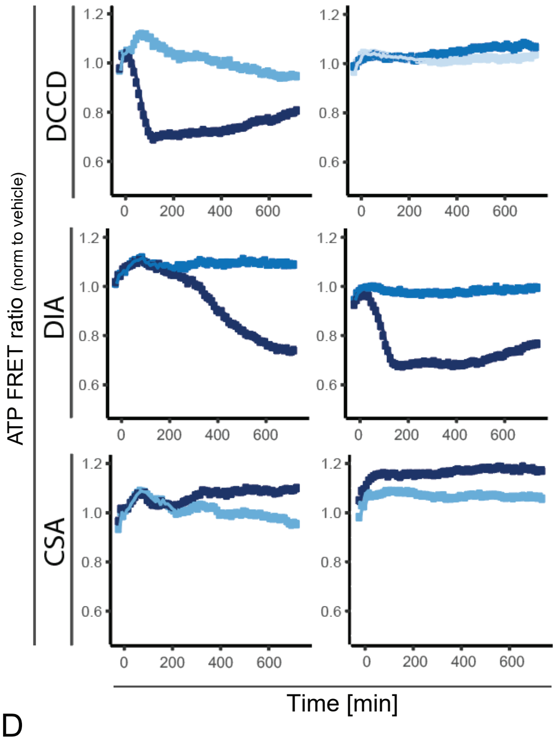

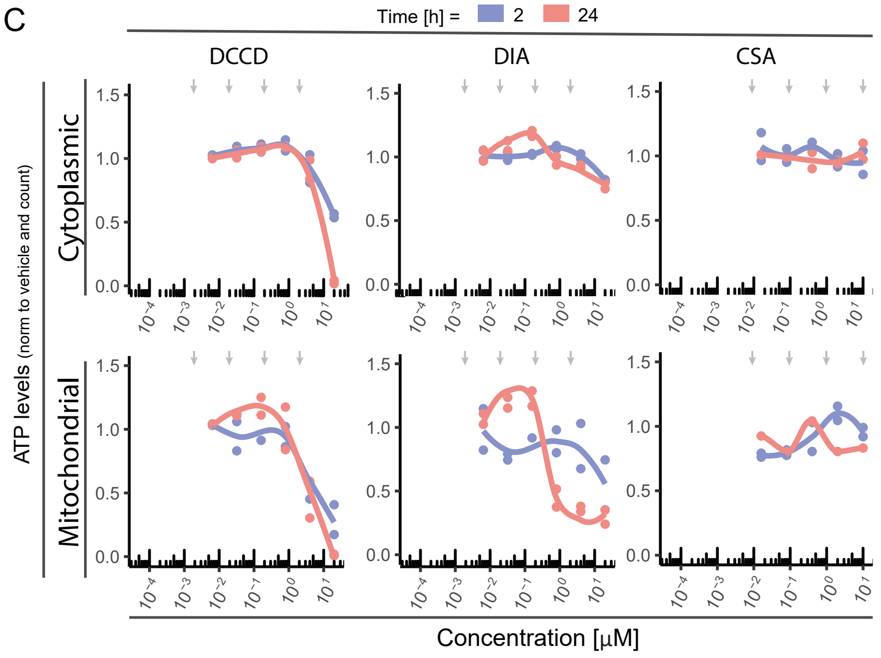

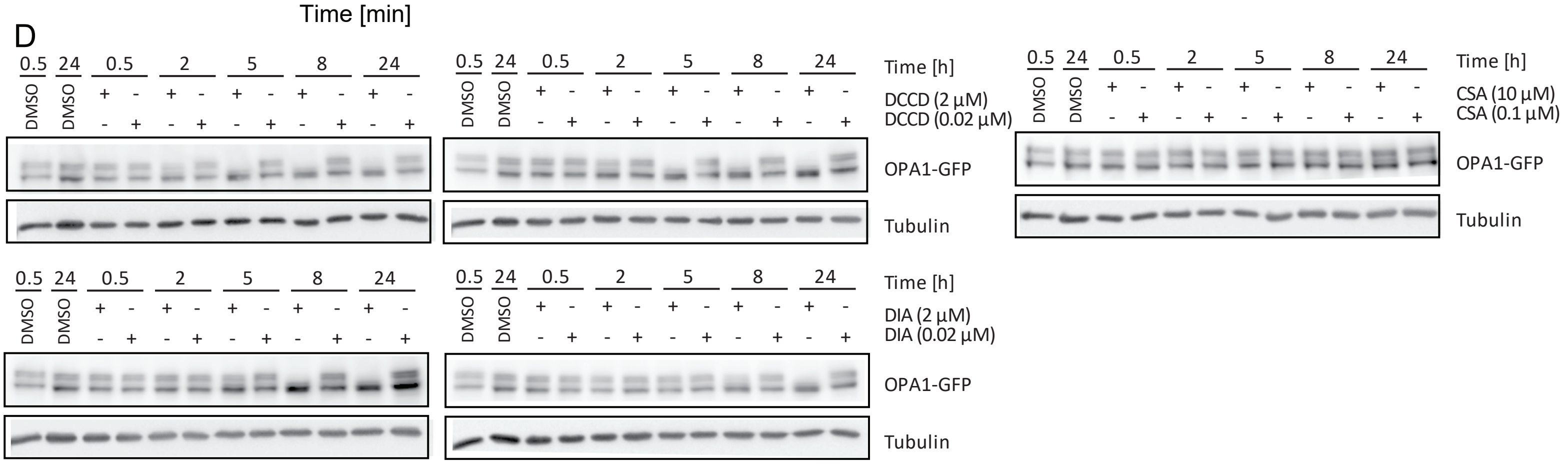

Supplementary Figure 5

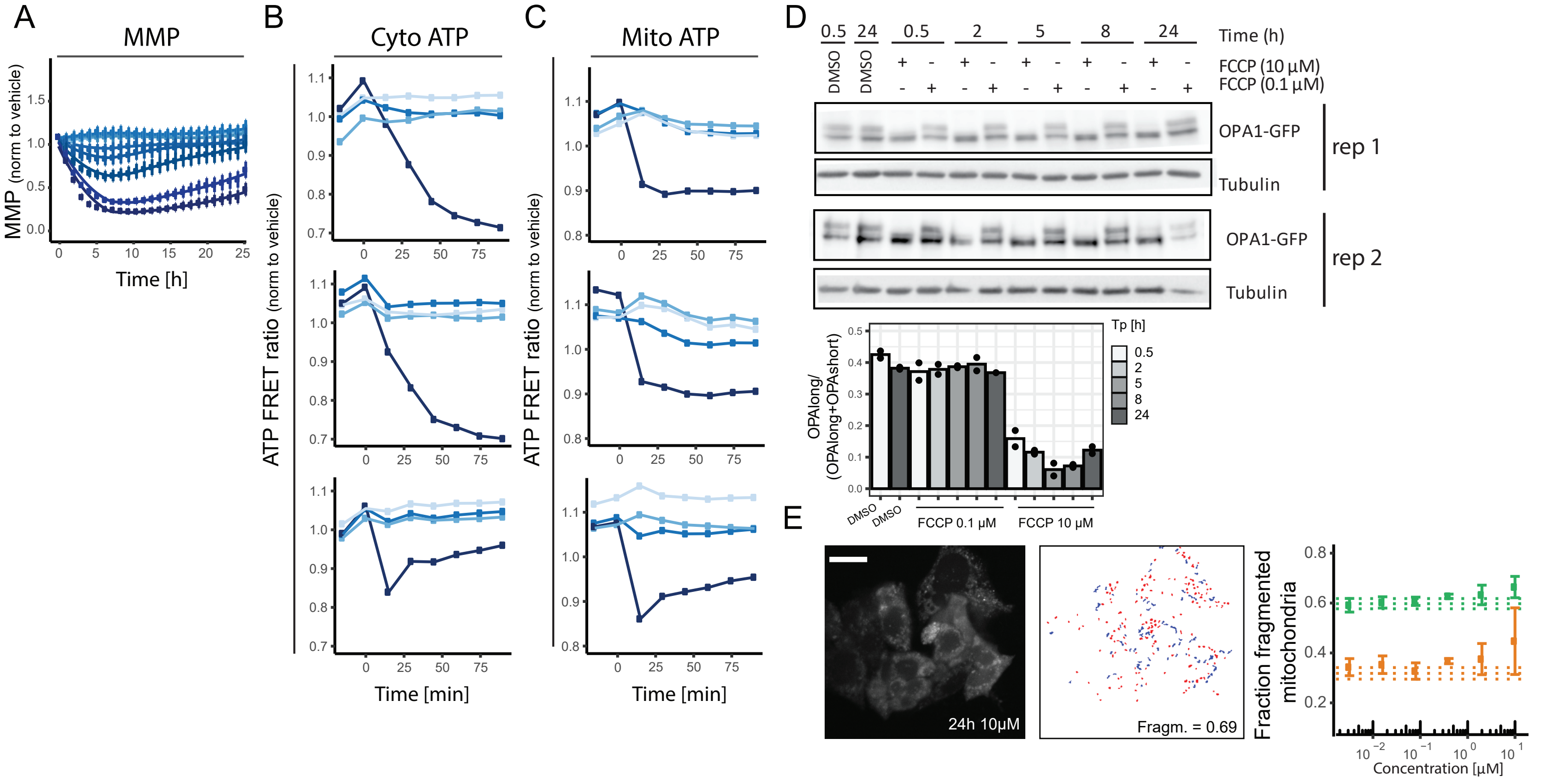

Mitochondrial membrane potential (A)

Concentration [µM] =

0.000128, 0.00064, 0.0032, 0.016, 0.08, 0.4, 1, 2, 5, 10, 25, 50

ATP biosensor (B&C)

Chemical	Abbr.	Concentration [µM]			
		4	3	2	1
FCCP	FCCP	5	0.5	0.05	0.005

Segmentation (E)

Classification
- fragmented
- fused

Nr. = Fragmented of total number (Vehicle =0.578)

Morphology (E)

Fraction Fragmented as:
- Fraction of Mitochondrial Number
- Fraction of: Mitochondrial Mass

Supplementary Figure 6

Compound	MoA	Morphology	MMP	ATPcyto	ATPmito	OPA1 cleavage
Rotenone	CI	=	↓	↓	↓	Slightly
Antimycin	CIII	=	↓	↓	↓	No
Oligomycin	CV	↑ fragmented	↑	↓	↓	Yes
DCCD	CV	↑ fragmented	↑	↓	↓	Yes
DIA	CV	↑ fragmented	↑	↓	↓	Yes
CSA	mPTP blocker	↑ fragmented / swelling	↑	=	=	No
FCCP	uncoupler	↑ fragmented	↓	↓	↓	Yes

Supplementary Figure 7
